# Specific Increase of Hippocampal Delta Oscillations Across Consecutive Treadmill Runs

**DOI:** 10.3389/fnbeh.2020.00101

**Published:** 2020-06-23

**Authors:** Alan M. B. Furtunato, Bruno Lobão-Soares, Adriano Bretanha Lopes Tort, Hindiael Belchior

**Affiliations:** ^1^Brain Institute, Federal University of Rio Grande do Norte, Natal, Brazil; ^2^Psychobiology Graduate Program, Federal University of Rio Grande do Norte, Natal, Brazil; ^3^Department of Biophysics and Pharmacology, Federal University of Rio Grande do Norte, Natal, Brazil; ^4^Faculty of Health Sciences of Trairí, Federal University of Rio Grande do Norte, Natal, Brazil; ^5^Center for Memory & Brain, Boston University, Boston, MA, United States

**Keywords:** hippocampal oscillations, delta, theta, gamma, locomotion speed, treadmill, running exercise

## Abstract

Running speed affects theta (6–10 Hz) oscillations, the most prominent rhythm in the rat hippocampus. Many reports have found a strong positive correlation between locomotion speed and the amplitude and frequency of theta oscillations. However, less is known about how other rhythms such as delta (0.5–4 Hz) and gamma (25–100 Hz) are affected, and how consecutive runs impact oscillatory activity in hippocampal networks. Here, we investigated whether the successive execution of short-term runs modulates local field potentials (LFPs) in the rat hippocampus. To do this, we trained Long-Evans rats to perform voluntary 15-s runs at 30 cm/s on a treadmill placed on the central stem of an eight-shape maze, in which they subsequently performed a spatial alternation task. We bilaterally recorded CA1 LFPs while rats executed at least 35 runs on the treadmill-maze apparatus. Within running periods, we observed progressive increases in delta band power along with decreases in the power of the theta and gamma bands across runs. Concurrently, the inter-hemispheric phase coherence in the delta band significantly increased, while in the theta and gamma bands exhibited no changes. Delta power and inter-hemispheric coherence correlated better with the trial number than with the actual running speed. We observed no significant differences in running speed, head direction, nor in spatial occupancy across runs. Our results thus show that consecutive treadmill runs at the same speed positively modulates the power and coherence of delta oscillations in the rat hippocampus.

## Introduction

The oscillatory activity in the rodent hippocampus expresses many different and sometimes coexisting frequency bands (Penttonen and Buzsáki, 2003; Buzsáki and Draguhn, 2004; Andersen et al., 2007; Tort et al., 2010). These rhythms are modulated by several aspects of the animal’s behavior, such as respiration (Yanovsky et al., 2014; Lockmann et al., 2016), cognition (McNaughton et al., 2006; Montgomery and Buzsáki, 2007; Belchior et al., 2014) and locomotion (Kuo et al., 2011; Ahmed and Mehta, 2012). Theta (6–10 Hz) oscillations are the most prominent hippocampal rhythm during locomotor behaviors (Buzsáki, 2002). Vanderwolf and Heron (1964) discovered that voluntary movements affect the magnitude of theta oscillations. Afterward, many reports have found a strong positive correlation between locomotion speed and the amplitude and peak frequency of theta oscillations (Vanderwolf, 1969, 1971; McFarland et al., 1975; Sławińska and Kasicki, 1998; Buzsáki, 2005; Long et al., 2014).

Hippocampal theta oscillations often occur concomitantly with faster rhythms in the gamma (25–100 Hz) band (Bragin et al., 1995; Csicsvari et al., 2003). Some reports have shown an inverse relationship between low gamma (25–55 Hz) and running speed in rats (Ahmed and Mehta, 2012; Kemere et al., 2013; Zheng et al., 2016; Lopes-dos-Santos et al., 2018) whereas studies in mice have shown a direct relationship (Chen et al., 2011; Lopes-dos-Santos et al., 2018). In turn, high gamma (65–100 Hz) power positively correlates with running speed (Chen et al., 2011; Ahmed and Mehta, 2012; Kemere et al., 2013; Zheng et al., 2016; Lopes-dos-Santos et al., 2018), following theta band power. Due to its prevalence in active behaviors, theta and gamma rhythms are believed to reflect on-demand processing (i.e., the processing of current information) in the hippocampus (Buzsáki, 2002; Colgin, 2013).

Conversely, during immobility and quiet behaviors, such as eating or grooming, hippocampal activity is dominated by a slower and irregular oscillation in the delta (0.5–4 Hz) frequency range (Andersen et al., 2007). The immobility to movement transition is accompanied by a decay of delta oscillations, and therefore the power in the delta band negatively correlates with locomotion and running speed (Schultheiss et al., 2019). In the cortex, delta power decreases along a long-term treadmill running at a constant speed of 21 cm/s (Li et al., 2008). Overall, the delta rhythm is often implicated in the regulation of autonomic functions, such as breathing and heartbeat (Knyazev, 2012), and delta band power is positively related to parasympathetic activity (Yang et al., 2003). The phases of delta oscillations allow the synchronization among subcortical nuclei and widespread cortical areas (Siapas and Wilson, 1998; Sirota et al., 2003; Sirota and Buzsáki, 2005; López-Azcárate et al., 2013), and may contribute to memory consolidation and other restorative functions of the slow-wave sleep (Buzsáki, 2002; Sirota et al., 2003; Tononi and Cirelli, 2006; Diekelmann and Born, 2010; Colgin, 2011; Rasch and Born, 2013).

Hence, hippocampal oscillatory activity is traditionally featured by two opposite and mutually exclusive states composed by theta and gamma rhythms or containing delta oscillations (Schultheiss et al., 2019), which are often used to, respectively, characterize “online” and “offline” modes of information processing (Buzsáki, 2002). In the present work, however, we present novel findings that challenge this traditional view. By investigating how the successive execution of short-term runs at the same speed affects oscillations in the rat hippocampus, we observed the concomitant occurrence of prominent delta and theta oscillations. While theta and gamma power significantly decreased, delta oscillations surprisingly emerged across consecutive runs. These changes were accompanied by a significant enhancement of the inter-hemispheric phase coherence in the delta band, but not in the theta and gamma bands. Our results thus show a specific increase in hippocampal delta oscillations across consecutive treadmill runs at the same speed, and highlight the effects of exercise over oscillatory changes in the rat hippocampus.

## Materials and Methods

### Animal Experimentation

All experimental procedures were approved by the Boston University Institutional Animal Care and Use Committee, and performed in strict accordance with the recommendations in the Guide for the Care and Use of Laboratory Animals of the National Institute of Health. Adult male Long-Evans rats (3–6 months old; weight among 400–550 g) were kept on a 12-hour light/dark cycle (lights on at 07:00 h), housed individually with free access to food and limited access to water, so as to maintain ∼85% of the body weight reached in *ad libitum* conditions. Animals were water deprived for 12-hour before experiments, and had free access to water for at least 3 h at the end of experiments and during the weekends.

### Apparatus and Training

**FIGURE 1:**
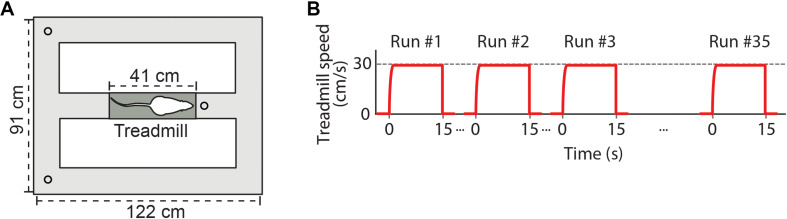
Experimental design. **(A)** Schematic representation of the apparatus showing the treadmill (dark gray) placed in the central stem of an eight-shape maze (light gray). Circles at each side of the maze and ahead of the treadmill denote ports to deliver drops of water. **(B)** The exercise protocol required rats to perform voluntary 15-s runs on the treadmill at a constant speed of 30 cm/s. Red lines represent instantaneous treadmill speed across subsequent runs. Inter-running intervals depended on the time the animal spent to complete a lap on the maze. Recording sessions with at least 35 runs on the treadmill were analyzed.

We used an electrical treadmill (Figure 1A; 41 cm × 12 cm, Columbus Instruments) placed in the central stem of an eight-shape maze (122 cm × 91 cm). Two ports for water delivery were located in the corners of the maze, and a third water port was located ahead of the treadmill. Water drops used as reward (approximately 0.05 ml) were automatically delivered according to the animal’s behavior and position on the treadmill-maze apparatus. Water reward was always delivered 1 s before each running start, when animals were positioned over the treadmill. A second water reward was delivered 1 s after animals performed an uninterrupted 15 s running. Animals were also rewarded after correct spatial alternations between the arms of the maze. Reward was delivered alternately in one water port at the corner of the maze depending on the last arm explored by the rat. Consecutive laps at the same maze arm were not rewarded at the corners’ water ports. Rewards at the treadmill water port were delivered independently of the animal’s performance on the spatial alternation task. Animals were behaviorally conditioned to perform voluntary treadmill runs to receive water reward, and no electrical shock was used on the apparatus. The treadmill activity and water delivery were controlled by an automated video tracking system (Cineplex, Plexon Inc.), which were based on the animal’s behavior on the treadmill (i.e., whether or not the animal performed a full 15-s running) and on the spatial trajectory since the last reward (i.e., whether or not the animals performed an alternated lap). The same desktop computer recorded behavioral, video and electrophysiological data, which were synchronized.

Animals were trained to execute voluntary 15-s runs at 30 cm/s on the treadmill (Figure 1B) in order to obtain a water reward. Before the beginning of the training, animals were individually handled for 5 min and introduced to the treadmill-maze apparatus for three consecutive days. In the habituation days, animals were allowed to freely explore the apparatus for 10–15 min with the treadmill turned off and water reward freely available at the three water ports. On the first day of training, every time the animals were located over the belt, water reward was delivered and the treadmill was automatically turned on at low speed (5 cm/s). The treadmill was turned off after 15 s or whenever the animals stop running. Treadmill speed was constant during each 15 s running, and maintained the same across all the runs in a given training session. Across subsequent training sessions, treadmill speed was gradually increased from 5 cm/s to 10, 15, 20, 25, and 30 cm/s according to the performance of individual animals, while the running duration was kept at 15 s. Animals were trained to reach a criterion of 35 runs at 30 cm/s for 15 s within three consecutive days.

### Electrodes and Surgery

After reaching the training criterion, animals were submitted to a stereotaxic surgery for the bilateral implant of 24 independently moving tetrodes targeting the CA1 area of the dorsal hippocampus (3.2 mm AP and 1.9 mm ML relative to Bregma; Paxinos and Watson, 1998). Tetrodes consisted of four twisted 12.7-micrometer polyimide-insulated nickel-chromium wires (Sandvik) electroplated in gold solution to reduce impedance to ∼200 kOhms at 1 kHz using NanoZ (Neuralynx). Tetrodes were assembled in a microdrive that allowed independent vertical movement. Each rat was anesthetized with isoflurane and placed in a stereotaxic apparatus (Kopf). The microdrive was chronically fixed over the parietal bones of the skull with dental cement. Electrical ground was provided by two stainless steel screws implanted in the occipital bones, ∼1–2 mm caudally to Lambda. Additionally, six stainless steel screws were used to mechanically support the microdrive. At the end of surgery, tetrodes were lowered approximately 1 mm into the brain tissue. After the surgical procedure, animals were allowed to recover for 7–10 days, and then tetrodes were slowly advanced toward the dorsal CA1 area of the hippocampus. Electrophysiological signals, such as the amplitude and phase of theta oscillations and sharp-wave/ripple complexes, and the presence of theta-modulated spikes, were used to guide tetrode positioning close to the pyramidal cell layer of CA1.

### Electrophysiological and Behavioral Recordings

Continuous electrophysiological and video recordings of animals were obtained during the execution of the treadmill-maze task. Experiments began daily at 13:00 h. A Multichannel Acquisition Processor (MAP, Plexon Inc.) was used for electrophysiological recordings. LFP signals were preamplified (1,000×), band-pass-filtered between 0.5 Hz and 400 Hz, and digitized at 1 kHz. Behavior was recorded by a digital video camera (30 frames per second) placed above the treadmill-maze apparatus. The instantaneous position and head direction of the animals were tracked through two LEDs placed at the head of the rats by an automated system (Cineplex, Plexon Inc.) which synchronized behavioral and neural data. Video and electrophysiological data were stored for posterior analysis.

### Histology

After the conclusion of experiments, the final position of the tetrode tips was confirmed by light microscopy of brain slices. Rats were overdosed with pentobarbital (100 mg/kg), and a 40 μA current was passed through each electrode for 30 s. Rats were then intracardially perfused with ferric ferrocyanide solution, followed by 4% paraformaldehyde solution. Brains were removed and stored in 4% paraformaldehyde/20% sucrose for 24 h and then frozen. Coronal brain sections (50 micrometers) were obtained in a cryostat (Microm), mounted on glass slides and stained with Cresyl Violet solution.

### Data Analysis

All analyses were performed using built-in and custom-made routines in MATLAB (MathWorks). One pair of contralateral electrodes from each session was selected for the following analysis. Electrode selection aimed to choose electrodes close to the CA1 pyramidal layer, and was based on electrophysiological features, such as the presence of sharp-wave ripples in the LFPs. Power spectral estimation was obtained by the Welch periodogram method using the “pwelch” function from the Signal Processing Toolbox (1 s window length with no overlap). The power in the delta (0.5–4 Hz), theta (6–10 Hz), low gamma (25–55 Hz) and high gamma (65–100 Hz) bands at each running were obtained through the averaged power spectrum within each frequency band. Power normalization was performed independently for each frequency band and before computing group results since different sessions had different levels of baseline power. We divided the power values of each running by a normalization factor defined as the mean power over the runs of the session under comparison. For instance, in Figure 3B, delta band power of each running was divided by the mean delta power over all the runs of the session. Therefore, the average of the normalized power values equals 1 in each session. The same normalization was used in coherence analysis. Inter-hemispheric phase coherence was estimated through the “mscohere” function from the Signal Processing Toolbox (50% overlapping Hamming windows with length of 2 s). The time-frequency decomposition was obtained by using the “spectrogram” function from the Signal Processing Toolbox (90% overlapping Hamming windows with length of 1 s). Spearman’s rank order correlations between (1) the mean band power and the trial number, and between (2) the mean inter-hemispheric band coherence and the trial number were performed using the “corr” function with the option “Type Spearman” from the Signal Processing Toolbox. Pearson’s linear correlations between (1) the mean band power and mean running speed, and between (2) the mean inter-hemispheric band coherence and the mean running speed of each individual treadmill running were performed using the “corr” function from the Signal Processing Toolbox.

The *X* and *Y* coordinates of two LEDs placed at the animal’s head were used to obtain the instantaneous position, locomotion speed and head direction of the animals. The instantaneous locomotion speed was defined as the distance traveled by the animals between consecutive video frames (30 frames/second). The instantaneous locomotion speed during treadmill running periods was added by the treadmill speed of 30 cm/s. The instantaneous head direction was defined as the angle formed by the alignment of the two LEDs and the horizontal plane along the treadmill (i.e., from left to right defined as 0 degrees). The latency to reward was defined as the time interval between the end of the treadmill running and the time animals reach the water ports at the corners of the maze.

### Statistical Analysis

Group results were expressed as mean ± SEM over sessions (*n* = 11). We used repeated measures ANOVA followed by Tukey-Kramer *post hoc* test to determine statistical difference between the means across the 35 runs. An alpha level of 0.01 was used to denote statistical significance (*p* < 0.01) in ANOVA, and 0.05 in the Spearman’s and Pearson’s correlation test (*p* < 0.05). All statistical analyses were performed in MATLAB.

## Results

**FIGURE 2:**
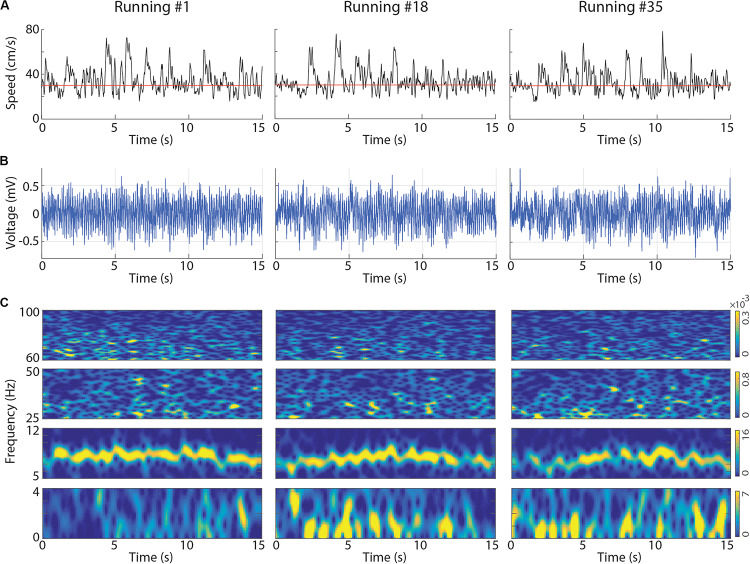
Representative examples of instantaneous running speed and LFP signals at the first, at an intermediate and at the last treadmill running. **(A)** Black lines represent the instantaneous running speed and red lines represent treadmill speed (30 cm/s). The CA1 LFPs **(B)** and associated spectrograms **(C)** obtained for the same runs. In the spectrograms, delta (0–4 Hz), theta (5–12 Hz), low gamma (25–50 Hz) and high gamma (60–100 Hz) bands are shown separately to allow individual colormap scales (the scale is the same across runs). Notice the gradual reduction of theta power and the emergence of a slower LFP activity in the delta band. LFPs obtained from the dorsal CA1 of the left hemisphere (Rat2_Session2).

We investigated whether successive executions of short-term treadmill running (Figure 1) modulate local field potentials (LFPs) in the rat hippocampus. We analyzed running speed and CA1 LFPs of 11 sessions recorded from three rats performing at least 35 voluntary 15-s runs at 30 cm/s on a treadmill. Visual inspection of the instantaneous running speed, of the raw LFP signals and their spectral decompositions revealed prominent activity in the theta band (6–10 Hz) and low levels of delta band (0.5–4 Hz) power during the first running (Figure 2, left panel), thus consistent with previous literature (Vanderwolf, 1969, 1971; McFarland et al., 1975; Sławińska and Kasicki, 1998; Buzsáki, 2005). However, consecutive runs at the same speed surprisingly exhibited a progressive decrease in theta power along with the emergence of bursts of delta activity, resulting in the concomitant occurrence of delta and theta waves (Figure 2C, center and right panels for runs number 18 and 35, respectively). The power in low gamma (25–55 Hz) and high gamma (65–100 Hz) bands followed changes in theta power. Notice in the example that the instantaneous running speed was not directly associated with changes in the power of the delta, theta and gamma bands (Figures 2A,C, respectively).

**FIGURE 3:**
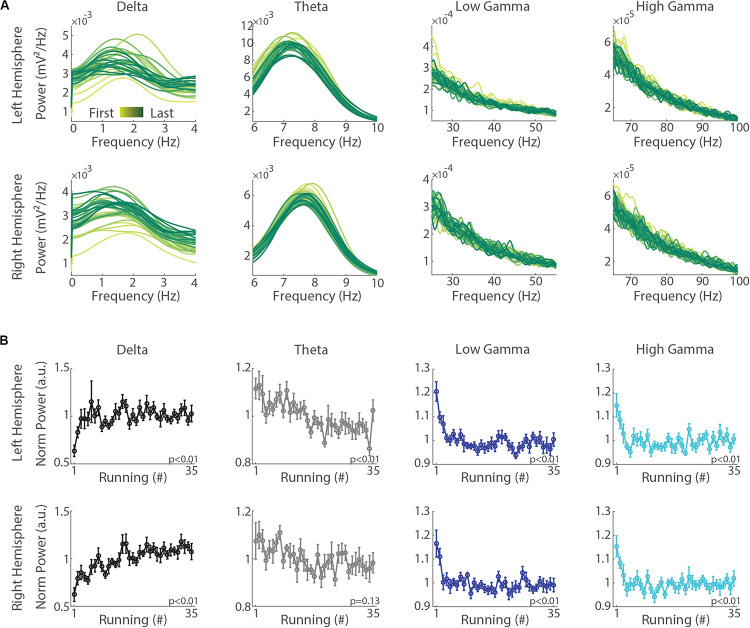
Consecutive treadmill runs specifically increase delta band power while decreasing the power in theta, low gamma and high gamma oscillations. **(A)** Mean power spectra across the 35 runs in the left (upper panels) and right (lower panels) hemispheres. Notice a gradual increase in delta band power concomitantly with a decrease in the power other bands from the first to the last running (yellow to green shades, respectively). **(B)** Normalized mean power of delta, theta, low gamma and high gamma oscillations along the successive execution of 35 runs in the left (upper panels) and right (lower panels) hemispheres. Circles represent means and error bars represent ± SEM (*n* = 11 sessions from three animals). The normalization was performed for each session by dividing power values on each running by the average across runs. Notice the significant increase of delta band power (*p* < 0.01, repeated measures ANOVA) along with significant decreases in the power of theta, low gamma and high gamma bands in both hemispheres (*p* < 0.01), except theta in the right hemisphere (*p* = 0.13).

To determine whether delta, theta and gamma oscillations change across the execution of consecutive treadmill runs at the same speed, we calculated their power in each of the 35 runs and averaged values across sessions (for individual sessions and means for individual animals, see Supplementary Figure S1). This analysis revealed a remarkable increase in delta band power across successive runs in both hemispheres (Figure 3A). Curiously, as opposed to delta, the mean power in the theta band decreased across runs, revealing a differential modulation between delta and theta. Consistently, when calculating the progression of the normalized mean band power across the 35 runs, we found a significant increase in the power of the delta oscillations and a significant decrease in the power of other frequency bands (Figure 3B, *p* < 0.01, repeated measures ANOVA).

**FIGURE 4:**
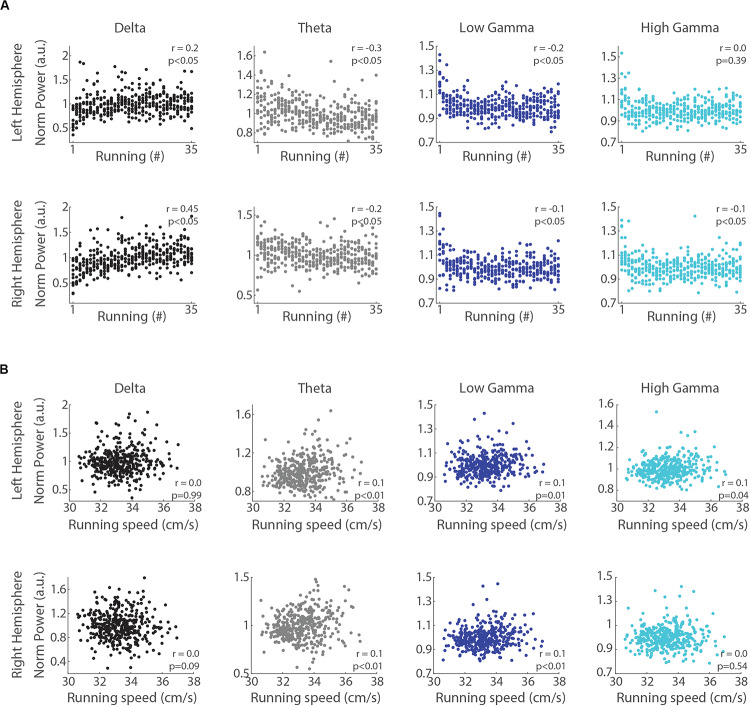
Delta power correlates with the trial number but not with running speed. **(A)** Normalized delta band power during treadmill running and the trial number (“running #”) directly correlated (*r* = 0.2, *p* < 0.05 and *r* = 0.45, *p* < 0.05, for left and right hemispheres, Spearman’s correlation), while the power of theta, low gamma and high gamma bands inversely correlated with the trial number (*p* < 0.05, Spearman’s correlation), except high gamma power in the left hemisphere (*p* = 0.39). **(B)** Normalized delta band power and running speed were not correlated (*p* = 0.99 and *p* = 0.09 for left and right hemispheres, respectively, Pearson’s correlation), while the power of theta, low gamma and high gamma bands weakly and positively correlated with running speed (*p* < 0.05, Pearson’s correlation), except high gamma power in the right hemisphere (*p* = 0.54).

We next analyzed whether LFP power in the delta, theta, low gamma and high gamma bands correlates with the trial number and the mean speed of each run (notice that even though the treadmill speed was constant, the animals could move back and forth along the belt). The Spearman’s rank order correlation revealed that delta power directly correlated with the trial number (Figure 4A, *r* = 0.2 and *r* = 0.45, *p* < 0.05, for left and right hemispheres, respectively). In contrast, the power of theta, low gamma and high gamma bands were inversely correlated (*r* = −0.3 and *r* = −0.2, *p* < 0.05, for theta in the left and right hemispheres; *r* = −0.2 and *r* = −0.1, *p* < 0.05, for low gamma in the left and right hemispheres; *r* = −0.1, *p* < 0.05, for high gamma in the right hemisphere), except for high gamma in the left hemisphere (*p* = 0.39). The Pearson’s linear correlation between delta band power and running speed showed no significant relationship (Figure 4B, *p* = 0.99 and *p* = 0.09 for left and right hemispheres, respectively), while the power in the theta, low gamma and high gamma bands weakly correlated with speed (*r* = 0.1, *p* < 0.05). These results indicate that consecutive runs promote specific increases in the power of delta oscillations that are dissociated from changes in running speed.

**FIGURE 5:**
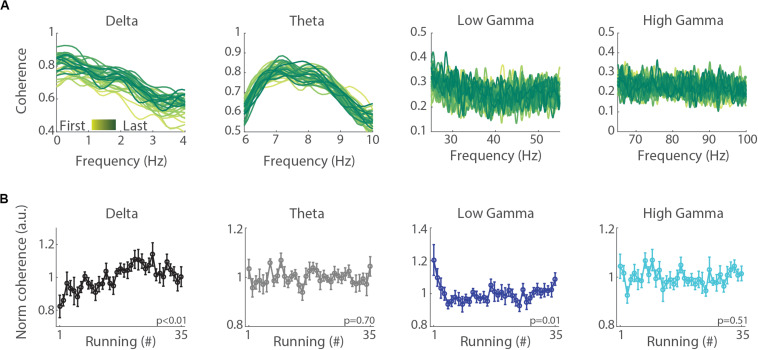
Inter-hemispheric delta coherence increases across treadmill runs. **(A)** Mean inter-hemispheric phase coherence in the delta, theta, low gamma and high gamma bands for each of the 35 runs. First to last runs are represented in lines colored from yellow to green shades. **(B)** Normalized mean inter-hemispheric coherence in the delta, theta, low gamma and high gamma bands. Circles denote means and error bars represent ± SEM (*n* = 11 sessions from three animals). The normalization was performed for each session by dividing coherence values on each running by the average across all the runs of the session. Notice that mean delta coherence significantly increases across runs (*p* < 0.01, repeated measures ANOVA), while mean theta and high gamma coherence presented no significant change (*p* = 0.70, *p* = 0.51, repeated measures ANOVA) and low gamma coherence tended toward a decrease (*p* = 0.01).

To evaluate whether the emergence of delta oscillations produces functional changes in the inter-hemispheric synchronization, we computed the LFP phase coherence between the left and right hemispheres. Figure 5A shows the mean spectral coherence across the 35 runs (see Supplementary Figure S2 for a typical session). We found that the mean inter-hemispheric coherence in the delta band gradually increased with consecutive runs (Figure 5B, *p* < 0.01), while the inter-hemispheric coherence in the theta and high gamma (65–100 Hz) bands did not change (*p* = 0.70, *p* = 0.51, respectively), and in the low gamma (25–55 Hz) band tended to decrease (*p* = 0.01).

**FIGURE 6:**
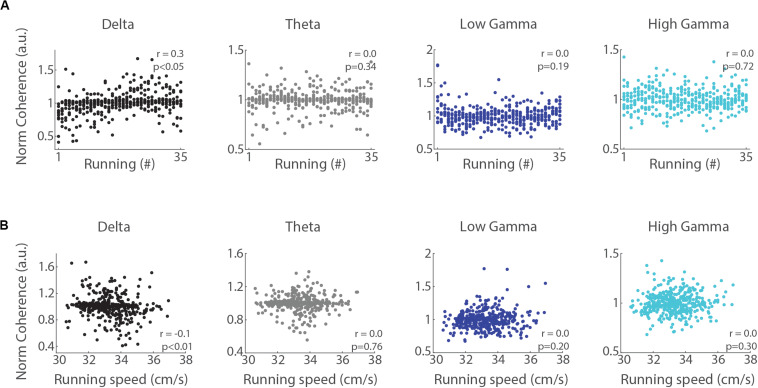
Inter-hemispheric delta coherence correlates with the trial number. **(A)** Normalized inter-hemispheric coherence in the delta band during treadmill running and the trial number (“running #”) moderately and positively correlated (*r* = 0.3, *p* < 0.05, Spearman’s correlation), while the other bands exhibited no significant relationship (*p* = 0.34, *p* = 0.19, and *p* = 0.72, for theta, low gamma and high gamma, respectively, Spearman’s correlation). **(B)** Normalized inter-hemispheric coherence in the delta band and running speed weakly and inversely correlated (*r* = –0.1, *p* < 0.05, Pearson’s correlation), while other bands exhibited no relationship (*p* = 0.76, *p* = 0.20, and *p* = 0.30, for theta, low gamma and high gamma, respectively, Pearson’s correlation).

We further analyzed whether the inter-hemispheric coherence during running correlates with the trial number and running speed. The Spearman’s rank order correlation revealed a significant positive relationship between coherence in the delta band and trial number (Figure 6A, *r* = 0.3, *p* < 0.05), but no significant correlation for the theta, low gamma and high gamma bands (*p* = 0.34, *p* = 0.19, and *p* = 0.72 for theta, low gamma and high gamma, respectively). Pearson’s linear correlation revealed a weak negative relationship between running speed and inter-hemispheric coherence in the delta band (Figure 6B, *r* = −0.1, *p* < 0.05). The inter-hemispheric coherence in theta, low gamma and high gamma bands did not correlate with running speed (*p* = 0.76, *p* = 0.20, and *p* = 0.30, respectively). These results suggest that delta may progressively coordinate network communication between both hippocampi across consecutive treadmill runs.

**FIGURE 7:**
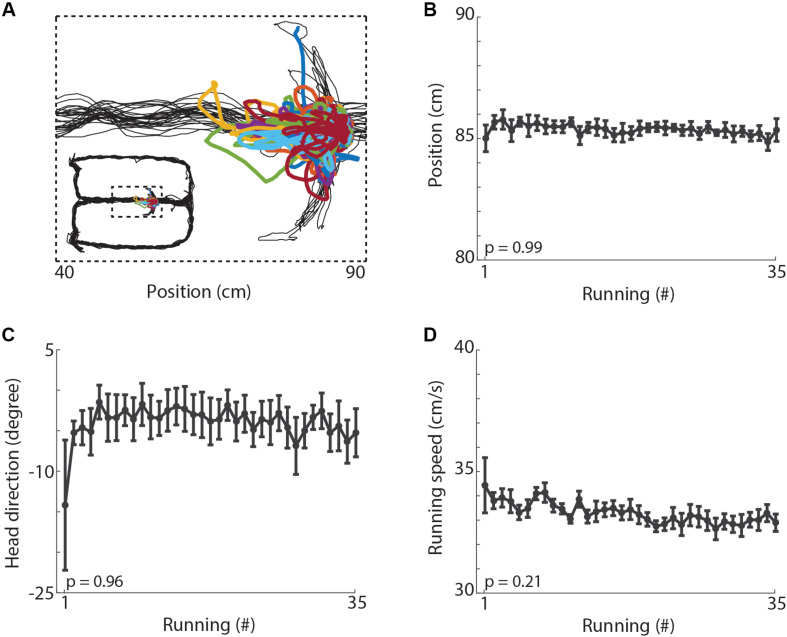
Behavioral variables. **(A)** Example of locomotion trajectories during maze laps (black lines) and treadmill runs (colored lines). **(B)** Mean position of the animals during treadmill runs across the 35 running periods (*p* = 0.99, repeated measures ANOVA). Circles denote mean and error bars indicate ± SEM (*n* = 11 sessions from 3 animals). **(C)** Mean head direction during treadmill running (*p* = 0.96, repeated measures ANOVA). The left-to-right treadmill axis is represented by zero degrees. **(D)** Mean running speed during each of treadmill run (*p* = 0.21, repeated measures ANOVA).

Finally, to investigate whether the changes observed in hippocampal oscillations could be explained by locomotor and behavioral variables, we evaluated the spatial position, head direction and locomotion speed during treadmill running. Figure 7A shows the trajectory of the animal along the maze and during treadmill runs in a typical recording session. We observed no significant changes in the average position (Figure 7B, *p* = 0.99), in the head direction (Figure 7C, *p* = 0.96), nor in the average locomotion speed (Figure 7D, *p* = 0.21) across the 35 runs. Additionally, we observed no difference in the latency from the end of the treadmill running until the time animals arrive at the reward location on the arms of the maze (Supplementary Figure S3A, *p* = 0.92). The rats usually spent less than 5 s at the water ports, the average inter-running interval was 43.68 s, and the mean session length was 33.46 min (see Supplementary Video S1). The ratio of correct spatial alternations over the 35 trials of the treadmill-maze task averaged 78%, ranging from 36 to 100% of correct choices (Supplementary Figure S3B). Thus, the locomotor and behavioral variables analyzed here do not account for the significant changes observed in hippocampal oscillations.

## Discussion

In this study, we analyzed the progression of hippocampal oscillations across 35 successive short-term (15 s) treadmill runs at the same speed (30 cm/s). Our results show that consecutive runs promote specific increases in the power of delta oscillations that simultaneously occur with decreases in theta and gamma oscillations (Figure 3). These changes were accompanied by the increase of inter-hemispheric phase coherence in the delta frequency range, with no significant changes of phase coherence in the theta and gamma bands (Figure 5). In addition, changes in band power and inter-hemispheric coherence were better correlated with the trial number than with running speed (Figures 4, 6). Our results suggest that delta and theta oscillations can coexist during locomotor behaviors, and that consecutive runs at the same speed differentially modulate delta, theta and gamma oscillations toward the progressive expression of the slowest rhythm in the rat hippocampus.

To the best of our knowledge, this is the first study to show the emergence of hippocampal delta oscillations while rats engage in active locomotor behavior and its simultaneous occurrence with theta oscillations. Hippocampal oscillations within the 0.5–4 Hz frequency range were first described as a large-amplitude irregular activity (Vanderwolf, 1969; Buzsáki and Draguhn, 2004; Andersen et al., 2007), in contrast to the prominent regular activity of theta oscillations. Most previous studies associate the hippocampal delta rhythm with immobility, quiet and consummatory behaviors or slow-wave sleep (Tobler and Borbély, 1986; Mölle and Born, 2011; Buzsáki, 2015), while during locomotor behaviors delta oscillations are reported as minor or absent (Li et al., 2008; Schultheiss et al., 2019).

We believe that previous research might have missed the increase of delta oscillations due to the remarkable prominence of theta oscillations in the hippocampus of rats engaged in active locomotion. In spite of that, preliminary evidence of speed-modulated delta oscillations was shown by Czurkó et al. (1999) and Molter et al. (2012). Czurkó et al. (1999) investigated the effects of locomotion speed on theta power and frequency and did not mention the strong increase in delta band power associated with higher running speeds of rats in a wheel, which is apparent in their Figure 5D. Similarly, Molter et al. (2012), reporting the modulation of theta power by slower rhythms, have also provided a preliminary indication that delta oscillations are progressively stronger as rats run faster on a wheel, but not in an open field or a maze (their Figure 3A). Consistent with Czurkó et al. (1999) and Molter et al. (2012), our results demonstrate the simultaneous expression of delta and theta oscillations while rats performed speed-controlled runs in a treadmill (Figure 2). These observations suggest revisiting the classical dichotomy between theta vs. delta brain states.

In addition, the results of Czurkó et al. (1999) and Molter et al. (2012) also suggest that delta oscillations are modulated by speed when rats run in a wheel. In the present study, however, the treadmill speed was constant (30 cm/s), the animals’ running speed did not statistically differ across runs (Figure 7D), and delta power did not correlate with running speed (Figure 4B). These findings show that the changes in delta power across consecutive runs dissociate from changes in running speed. Based on the studies of Czurkó et al. (1999) and Molter et al. (2012), however, we could expect that consecutive runs at faster speeds (e.g., 50 cm/s) would promote even larger increases in delta power than found here. Further studies that analyze hippocampal oscillations while rats perform treadmill runs at different speeds will be necessary to characterize the speed-modulation of delta band power.

The major causes underlying the increase of delta oscillations across consecutive runs remain to be determined. Recent reports have pointed to multiple factors affecting oscillatory activity in the delta band. For instance, it was recently discovered that respiratory rhythms entrain hippocampal oscillations in the 1–4 Hz frequency range (Yanovsky et al., 2014; Lockmann et al., 2016; Nguyen Chi et al., 2016). Our study does not rule out the contribution of changes in respiratory entrainment to the increase of delta oscillations across runs. However, rats engaged in active locomotion usually breathe at higher frequencies around 8–10 Hz (Whishaw and Kolb, 2005; Kabir et al., 2010; Nalivaiko et al., 2010; Wachowiak, 2011; Tort et al., 2018), that is, at the theta frequency range. Therefore, further studies that simultaneously monitor respiratory rhythms along with hippocampal LFPs during treadmill running are required to investigate their potential relationship.

Alternatively, we propose that the increase of hippocampal delta power and inter-hemispheric coherence across successive runs could reflect the development of fatigue. The role of neuronal oscillations underlying the mechanisms of central fatigue remains unclear, but the signaling of fatigue in the brain may take place even before any reduction in performance outputs (Davis and Bailey, 1997; Lohnas et al., 2015; Cheron et al., 2016; Tran et al., 2020). According to this view, the brain processes that support the maintenance of running exercise cannot be sustained indefinitely but depend on neural resources that are progressively depleted along periods of high activity (Tulving and Rosenbaum, 2006; Poldrack, 2015). Thus, delta oscillations or its inter-hemispheric coherence could signal the progressive depletion of neural resources. Above a given threshold, additional increases in delta power would lead to a decrease in performance and eventually to exercise interruption. Interestingly, previous studies showed that prolonged awake state caused by sleep deprivation increases oscillations in the 2–6 Hz frequency range, consistent with a brain signaling of fatigue (Vyazovskiy et al., 2011). Although our experiments were not designed to study the mechanisms of central fatigue associated with exercise, they provide preliminary clues. Further studies that analyze hippocampal oscillations and markers of fatigue should be able to address this question.

In contrast to the increase of delta power, we found a significant reduction in the power of theta and gamma oscillations across runs, indicating that consecutive running exercise inversely affects these bands. This could reflect a general reduction in neuronal excitability, promoting shifts in the network oscillatory dynamics from faster to slower frequencies (Buzsáki and Draguhn, 2004). These results corroborate previous reports suggesting that hippocampal theta and delta rhythms may compete for the expression of oscillatory activity in the rat hippocampus, but also challenges the traditional view that the occurrence of delta and theta oscillations is mutually exclusive (Buzsáki et al., 1983; Buzsáki, 2002; Andersen et al., 2007; Schultheiss et al., 2019).

Classically, theta oscillations in the rat hippocampus have been associated with preparatory movements and locomotor behavior (Vanderwolf and Heron, 1964). Many previous studies have reported that theta oscillations strongly correlate with locomotion speed (Vanderwolf, 1969, 1971; McFarland et al., 1975; Sławińska and Kasicki, 1998; Buzsáki, 2005; Hinman et al., 2011; Long et al., 2014), while others have suggested that theta oscillations are not purely associated to locomotion speed but better reflect movement intensity in general (Whishaw and Vanderwolf, 1973; Bland et al., 2006; Tanaka et al., 2018). Since treadmill speed was always the same in our experiment (30 cm/s), according to the locomotion speed/movement intensity hypothesis, one would expect no changes in theta power across subsequent runs. However, we surprisingly found a significant decrease in theta power that cannot be explained by changes in running speed. Therefore, our results are contrary to this hypothesis, and the decrease in theta power may be associated with other physiological changes taking place across consecutive runs.

Previous studies have shown that motivational states affect hippocampal theta oscillations (Sławińska and Kasicki, 1998; Tracy et al., 2001). Here, the task design required water-deprived animals to perform voluntary treadmill runs in order to receive drops of water reward (no electrical shock was delivered at the treadmill; the task was solely based on appetitive behavior). Thus, after the animals have performed a few trials and repeatedly obtained a certain amount of water, their internal drive to perform the task naturally decreases as a consequence of satiety signals (Mogenson et al., 1980; Wise, 2004; Salamone and Correa, 2012). Therefore, another explanation for the decrease in theta power would be a reduction in the animals’ motivation to perform the task.

A third hypothesis comes from pioneer studies in which the theta rhythm has been related to general arousal and neuronal excitability (Green and Arduini, 1954). Arousal-related theta oscillations (also called type 2 theta) are usually greater when animals are alert to sensorial stimuli but often decay after repeated presentation of the same innocuous stimuli, a process generally known as habituation (Kay, 2005; Andersen et al., 2007; Kumaran and Maguire, 2009; Hinman et al., 2011). In the present study, the progressive reduction in theta band power could reflect a reduction in alertness and neuronal excitability due to the task habituation. Note that the consecutive runs had the same running speed, duration, distance traveled, and the same environmental conditions with constant sensorial cues, which is to say that animals were repeatedly exposed to the same set of stimuli.

Finally, we have proposed above that the changes in delta power observed here could signal the development of fatigue. Complementary to this hypothesis, the reduction in theta and gamma band power could also be related to the neural resources available to perform the running exercise (Tulving and Rosenbaum, 2006; Lohnas et al., 2015; Poldrack, 2015; Cheron et al., 2016; Tran et al., 2020). In this way, reductions of theta and gamma power would indicate a decrease in the internal readiness to perform the motor act.

Since the habituation hypothesis fundamentally contradicts the fatigue hypothesis, future experiments could approach this question by recording hippocampal LFPs while animals perform blocks of consecutive runs under a given constant speed (for instance, 10 runs at 30 cm/s) followed by blocks at higher running speed (10 runs at 40 cm/s). Based on the habituation hypothesis, one would expect a decay in theta power across the first 10 runs due to habituation to the repeated exposure to the same stimulus. Then, at the beginning of the second block of runs, theta power would exhibit a similar magnitude as at the beginning of the first block, revealing increased excitability triggered by the new stimulus intensity. Alternatively, however, if this experiment reveals a further reduction in theta power, it would in turn indicate that theta is progressively affected by the development of fatigue. Moreover, considering instead that the second block of runs reduces running speed (such as from 30 to 20 cm/s), based on the habituation hypothesis one would expect increases in theta power caused by novelty-related excitability, and based on the fatigue hypothesis, one would expect that theta power continues to decrease though in a less accentuated manner.

Our study used an animal model to investigate how successive runs affect oscillatory electrophysiological activity in the hippocampus, a deep structure in the medial temporal lobe, traditionally associated with learning and memory functions in humans and other animals (Scoville and Milner, 1957; Eichenbaum, 2002; Buzsáki and Moser, 2013). Since deep brain electrophysiological recordings in humans are scarce (not to mention the ethical implications and significant limitations to study exercise in implanted subjects), very few is known about how the human hippocampus responds to locomotion (see Watrous et al., 2011). From a translational point of view, a better understanding of the effects of running exercise over the hippocampus could be applied to improve exercise prescription in neurological patients, such as those suffering from memory deficits in Alzheimer disease (Radak et al., 2010; Intlekofer and Cotman, 2013). More specifically, a better knowledge of hippocampal rhythms during exercise can potentially contribute to the development of neurofeedback techniques applied in clinical conditions and in sports (see di Fronso et al., 2016).

Some limitations of the present study should be taken into account. First, despite undergoing the same training protocol, the number of days to reach the criterion (to perform 35 runs at 30 cm/s for 15 s in three consecutive days) differed among rats. This is important because the same speed could represent different exercise intensities for different animals, and because different animals could be differently conditioned to the task. A second limitation is that we did not define an *a priori* criterion for exercise-related fatigue. We analyzed the same number of runs for all animals and excluded any additional running after the 35th. It is possible that in some sessions the animals were exhausted at the 35th run, limiting a proper interpretation of fatigue in our experiments. Third, we analyzed a pool of 11 sessions recorded from three rats executing the task. The number of animals is thus low, though we verified that the results were qualitatively similar in all the rats and that no single animal was driving the group results. Fourth, we did not analyze potential LFP changes related to behavioral or cognitive demands of the spatial alternation memory task, i.e., left versus right turns or correct versus incorrect alternations. Finally, we only analyzed the power and coherence of hippocampal oscillations, but did not investigate whether the consecutive running exercise also affects spiking activity. Future studies should address these and other open questions.

We conclude that consecutive treadmill runs promote gradual and specific increases of delta oscillations in the rat hippocampus, and that theta and delta oscillations can coexist during locomotor behavior. We suggest that oscillatory changes toward slower frequencies may be required to cope with changes in internal physiological states during performance of running exercise.

## Data Availability Statement

The datasets generated for this study are available on request to the corresponding author.

## Ethics Statement

The animal study was reviewed and approved by the Boston University Institutional Animal Care and Use Committee.

## Author Contributions

HB designed the research and performed the experiments. AF and HB analyzed the data. All authors wrote the manuscript and contributed to the article and approved the submitted version.

## Conflict of Interest

The authors declare that the research was conducted in the absence of any commercial or financial relationships that could be construed as a potential conflict of interest.

## Supplementary Material

FIGURE S1 **(A)** Mean absolute (i.e., non-normalized) power in the delta and theta bands for individual sessions. Upper, middle and lower panels show data from Rat2_Session1, Rat3_Session1, and Rat11_Session2, respectively. **(B)** Average absolute power in the delta and theta bands across all sessions of each rat. Upper, middle and lower panels show data from Rat2_Session1, Rat3_Session1, and Rat11_Session2, respectively. Only data from the left hemispheres are shown.

FIGURE S2 **(A)** Inter-hemispheric phase coherence of 35 individual runs (red) and mean coherence of the session (black, Rat3_Session10). **(B)** Mean inter-hemispheric phase coherence in the delta (upper) and theta (lower) bands across 35 runs of the same session. **(C)** Mean inter-hemispheric phase coherence over 385 runs from 11 sessions (solid line). Dashed lines depict ± SEM.

FIGURE S3 **(A)** Latency from the end of treadmill running to the moment animals get the water reward at the maze corners (*p* = 0.92, repeated measures ANOVA). Circles represent means and error bars represent ± SEM (*n* = 11 sessions from 3 rats). The mean latency of each session is represented in colored lines. **(B)** Percentage of correct alternations across the 35 runs (*n* = 11 sessions from 3 rats).

VIDEO S1 Representative example of the treadmill-maze task. The video shows two consecutive laps on the apparatus during a typical recording session. Notice that the rat alternates among the left and right arms of the maze.
