# Supplementary material for: Specific Increase of Hippocampal Delta Oscillations Across Consecutive Treadmill Runs
Source: Front Behav Neurosci. 2020 Jun 23;14:101. doi: 10.3389/fnbeh.2020.00101 (PMC7333663; doi:10.3389/fnbeh.2020.00101)
Supplement: FIGURE S1 — (A) Mean absolute (i.e., non-normalized) power in the delta and theta bands for individual sessions. Upper, middle and lower panels show data from Rat2_Session1, Rat3_Session1, and Rat11_Session2, respectively. (B) Average absolute power in the delta and theta bands across all sessions of each rat. Upper, middle and lower panels show data from Rat2_Session1, Rat3_Session1, and Rat11_Session2, respectively. Only data from the left hemispheres are shown. [file Data_Sheet_1.PDF]

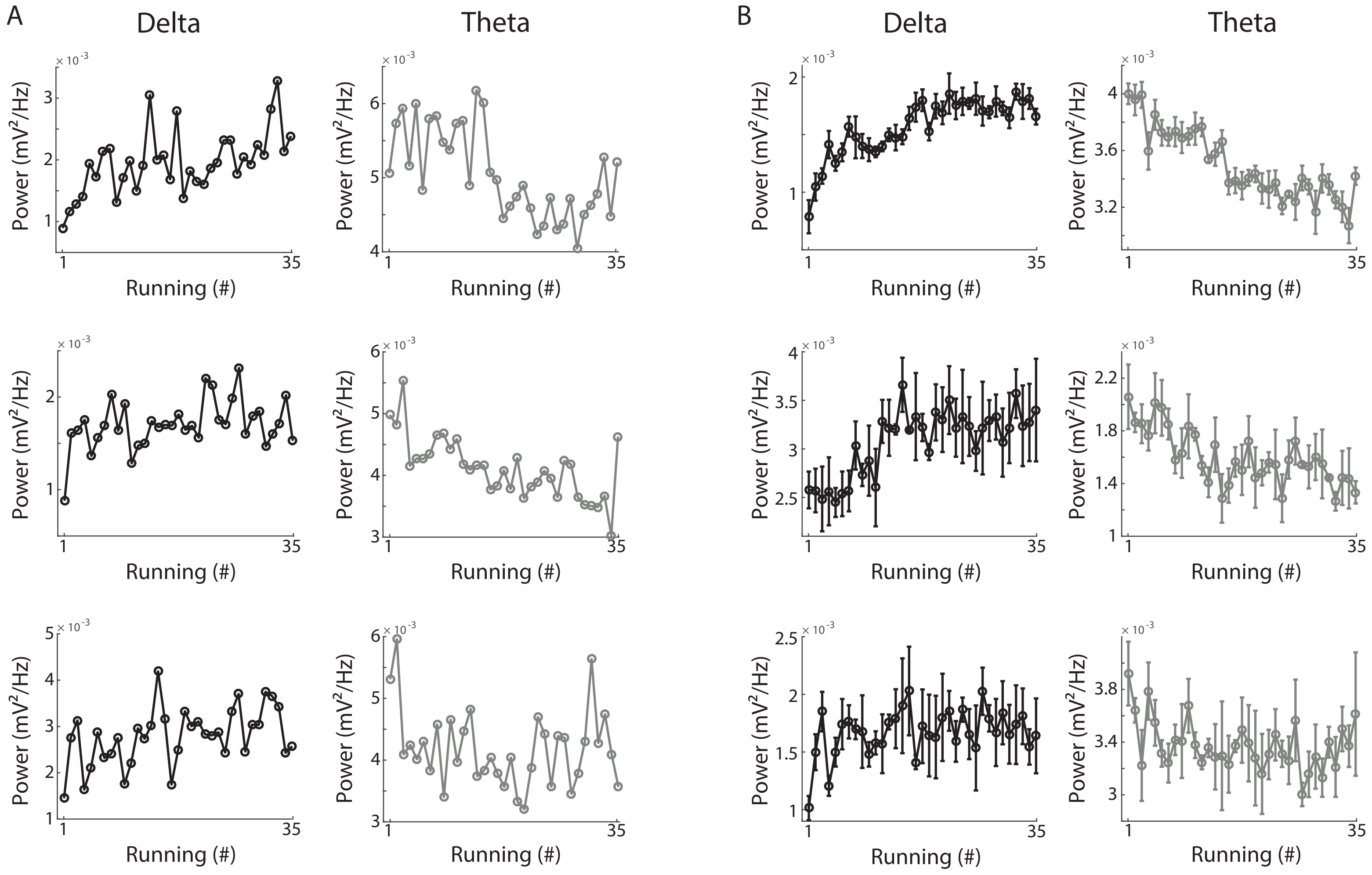


SUPPLEMENTARY FIGURE 1 | (A) Mean absolute (i.e, non-normalized) power in the delta and theta bands for individual sessions. Upper, middle and lower panels show data from Rat2_Session1, Rat3_Session1 and Rat11_Session2, respectively.
(B) Average absolute power in the delta and theta bands across all sessions of each rat. Upper, middle and lower panels show data from Rat2_Session1, Rat3_Session1 and Rat11_Session2, respectively. Only data from the left hemispheres are shown.

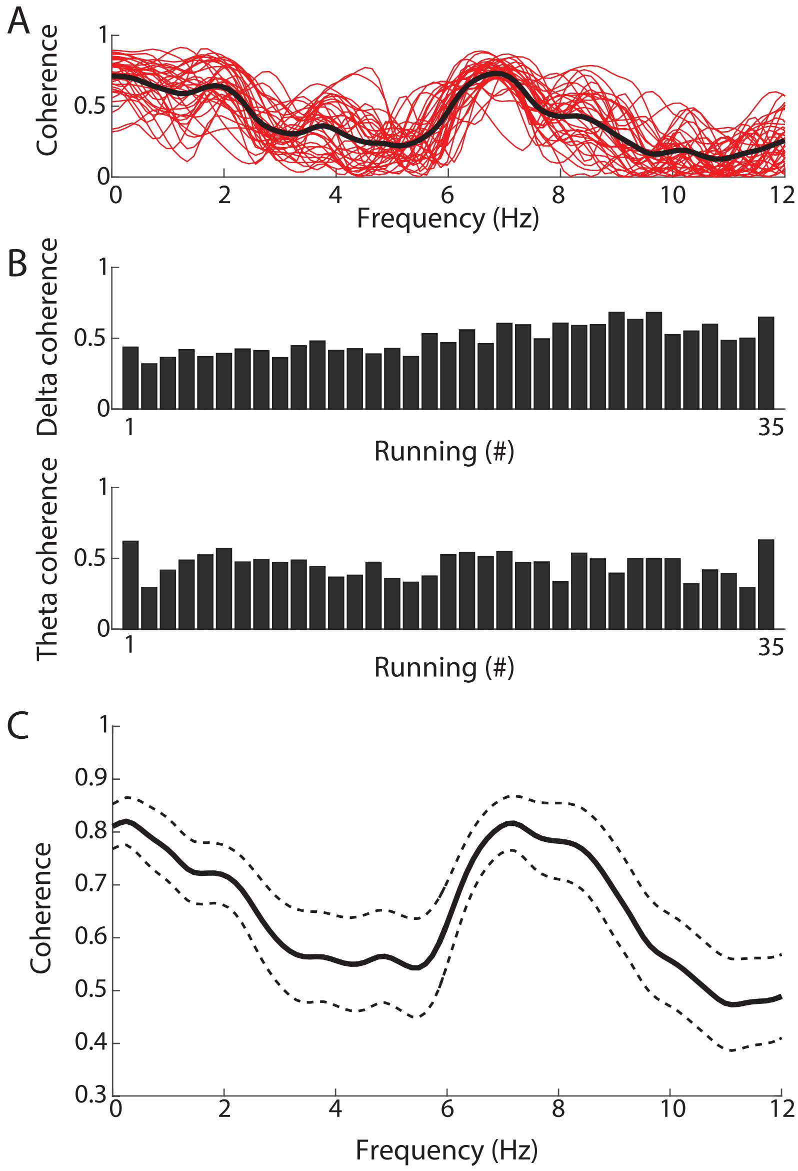


SUPPLEMENTARY FIGURE 2 | (A) Inter-hemispheric phase coherence of 35 individual runs (red) and mean coherence of the session (black, Rat3_Session10). (B) Mean inter-hemispheric phase coherence in the delta (upper) and theta (lower) bands across 35 runs of the same session. (C) Mean inter-hemispheric phase coherence over 385 runs from 11 sessions (solid line). Dashed lines depict ± SEM.

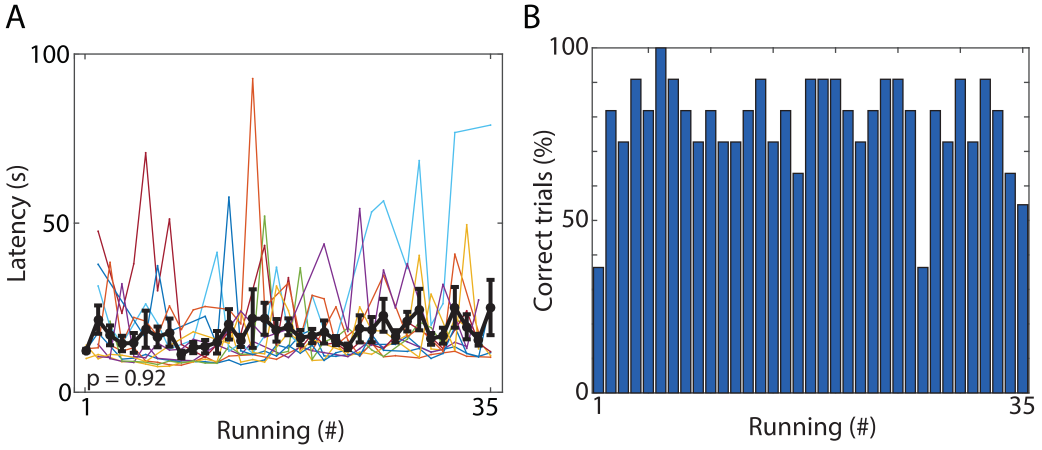


SUPPLEMENTARY FIGURE 3 | (A) Latency from the end of treadmill running to the moment animals get the water reward at the maze corners (p=0.92, repeated measures ANOVA). Circles represent means and error bars represent ± SEM (n=11 sessions from 3 rats). The mean latency of each session is represented in colored lines. (B) Percentage of correct alternations across the 35 runs (n=11 sessions from 3 rats).
